# Stores Healthy Options Project in Remote Indigenous Communities (SHOP@RIC): a protocol of a randomised trial promoting healthy food and beverage purchases through price discounts and in-store nutrition education

**DOI:** 10.1186/1471-2458-13-744

**Published:** 2013-08-12

**Authors:** Julie Brimblecombe, Megan Ferguson, Selma C Liberato, Kylie Ball, Marjory L Moodie, Anne Magnus, Edward Miles, Amanda J Leach, Mark D Chatfield, Cliona Ni Mhurchu, Kerin O’Dea, Ross S Bailie

**Affiliations:** 1 Menzies School of Health Research, Building 58, Royal Darwin Hospital Campus, Rocklands Drive, Tiwi, NT 0810, Australia; 2 Charles Darwin University, Ellengowan Dr, Casuarina, NT 0810, Australia; 3 University of South Australia, Frome Rd, Adelaide, SA 5000, Australia; 4 Centre for Physical Activity and Nutrition Research, Deakin University, Burwood Hwy, Burwood 3125, Australia; 5 Deakin Health Economics Faculty of Health, Deakin University, 221 Burwood Hwy, Burwood 3125, Australia; 6 Indigenous Community Volunteers Level 1, 29 Cavanagh St, Darwin, NT 0801, Australia; 7 National Institute for Health Innovation, University of Auckland, Auckland, New Zealand

**Keywords:** Price discount, Nutrition education, Randomised multiple baseline, Aboriginal Australia

## Abstract

**Background:**

Indigenous Australians suffer a disproportionate burden of preventable chronic disease compared to their non-Indigenous counterparts – much of it diet-related. Increasing fruit and vegetable intakes and reducing sugar-sweetened soft-drink consumption can reduce the risk of preventable chronic disease. There is evidence from some general population studies that subsidising healthier foods can modify dietary behaviour. There is little such evidence relating specifically to socio-economically disadvantaged populations, even though dietary behaviour in such populations is arguably more likely to be susceptible to such interventions.

This study aims to assess the impact and cost-effectiveness of a price discount intervention with or without an in-store nutrition education intervention on purchases of fruit, vegetables, water and diet soft-drinks among remote Indigenous communities.

**Methods/Design:**

We will utilise a randomised multiple baseline (stepped wedge) design involving 20 communities in remote Indigenous Australia. The study will be conducted in partnership with two store associations and twenty Indigenous store boards. Communities will be randomised to either i) a 20% price discount on fruit, vegetables, water and diet soft-drinks; or ii) a combined price discount and in-store nutrition education strategy. These interventions will be initiated, at one of five possible time-points, spaced two-months apart. Weekly point-of-sale data will be collected from each community store before, during, and for six months after the six-month intervention period to measure impact on purchasing of discounted food and drinks. Data on physical, social and economic factors influencing weekly store sales will be collected in order to identify important covariates. Intervention fidelity and mediators of behaviour change will also be assessed.

**Discussion:**

This study will provide original evidence on the effectiveness and cost-effectiveness of price discounts with or without an in-store nutrition education intervention on food and drink purchasing among a socio-economically disadvantaged population in a real-life setting.

**Trial registration:**

Australian New Zealand Clinical Trials Registry: ACTRN12613000694718

## Background

Indigenous people living in very remote communities in Australia have the poorest health outcomes of any population group in high income countries [1,2]. This burden of disease is in a large part due to poor nutrition across the life-course and is causing serious illness in what should be the most productive years of life. High food costs and generally low socio-economic status support a diet characteristically high in refined cereals, sugar and low in fruit and vegetables [3]. While efforts by individual communities and the Australian government to address food affordability and improve diet in this setting continue, there is little evidence on the effectiveness of different strategies.

There is compelling evidence that increasing fruit and vegetable intake and reducing sugar-sweetened soft-drink consumption can reduce the risk of preventable chronic disease. It has been estimated globally that an increase of 80 g per day of fruit and vegetables could reduce the risk of ischaemic heart disease by 10%, ischaemic stroke by 6%, lung cancer by 4% and oesophageal cancer by 6% [4]. Such an increase in fruit and vegetable intake in Australia could save approximately AUD157 million per year from the costs of cardiovascular disease (CVD) alone [5]. The benefits for the Indigenous population living in remote Australia are likely to be much greater considering their currently low intake of fruit and vegetables [3] and high burden of disease [1].

We have reported high consumption of sugar-sweetened soft-drinks in three remote communities of 474 ml (SD 51) per person per day on average [3]. Consumption of ≥ 1 serve of sugar-sweetened soft-drink per day (360 ml) has been associated with a higher prevalence of obesity among middle-aged women [6] and a reduction of one serving per day has been associated with a weight loss of approximately 0.5 kg at six and 18 months [7]. There is also the possibility that high fructose (the major sugar in many sugar-sweetened soft-drinks) intake is a direct risk factor for metabolic syndrome and Type 2 diabetes [8], which are highly prevalent in remote Australian Indigenous populations [9,10].

This evidence indicates that gains in health outcomes for Indigenous Australians living in remote communities could result from a shift in current consumption patterns, particularly in fruit and vegetables and sugar-sweetened soft-drink. There is growing evidence that taxes and discounts on food and beverages could influence consumption patterns considerably, and thus improve health outcomes [11-14]. Improved affordability has been associated with significant increases in the purchase and consumption of healthier foods [14,15].

Evidence from price discount studies suggests that demand for fruit-and-vegetables is price elastic – a 1% decrease in price is associated with more than a 1% increase in demand [14]. Waterlander et al. showed that a 50% discount on fruit and vegetables increased fruit and vegetable purchasing by a factor of four [16] and Ni Mhurchu et al. in a study conducted with supermarkets in New Zealand (NZ), showed a 10% increase in fruit and vegetable purchasing with a 12.5% price discount on healthy food options (the equivalent of removal of the NZ Goods and Services Tax) [13]. An et al. reported that 10% and 25% discounts on healthier food purchases were associated with an increase in daily fruit/ vegetable intake by 0.38 and 0.64 servings respectively [11]. There is little such evidence for socio-economically disadvantaged populations. The one study to date that has examined the differential price discount on food purchases across socio-economic groups, showed no variation in intervention effect by household income or education [17].

No studies, other than the SHELf study that is currently being conducted in Melbourne, Australia, have examined the cost effectiveness of price discount interventions [18] and little is known about the impact of subsidies on the overall diet and possible unintended consequences on diet[14]. A study conducted among urban poor households in two provinces in China demonstrated that reducing the price of a staple food commodity did not lead to an increase in demand for that commodity as people were able to purchase other foods that were highly valued but not necessarily of higher nutritional value [19]. This may not be the case however for households in high income countries. Ni Mhurchu et al., reported no change in purchase of less healthy foods and overall expenditure with a price discount on healthy foods [13]. Similarly Waterlander et al., reported no change in total expenditure, expenditure on non-food items or non-fruit and vegetable food items with a 50% price reduction on fruit and vegetables [16]. This uncertainty and lack of evidence however has impacted on policy adoption of economic incentive to improve diet [14].

It is posited that intervention to improve population diet needs to involve approaches that both target the individual and create an environment that supports individual food choice [20]. Nutrition education (targeting the individual) is frequently used to encourage healthy food purchasing, however the evidence for nutrition education impacting on healthy food purchasing is largely inconclusive [14]. Waterlander et al. showed no effect on fruit and vegetable purchasing with nutrition education alone (i.e., through provision of recipe books and telephone counselling) but reported an added beneficial effect when combined with a 50% price discount [16]. Ni Mhurchu et al. reported that tailored nutrition education, that suggested substitution of purchased unhealthy foods with specific healthier options, had no evident effect on the amount of purchased foods [13]. An et al. reported mixed results for nutrition education strategies using product labelling, promotional signage, stimulation (i.e., a text to remind/encourage action) or health messages (i.e., a text message to introduce health benefit of nutritious food intake) [14]. The benefit of combining a skill building nutrition education intervention (that develops budgeting, shopping and cooking skills), with a price discount, is being investigated as a means to improve dietary intake by the SHELf study in Melbourne, Australia [18].

Although consumers globally are driven by price, taste and convenience [21], the context of remote Australian communities is distinctive in the array of geographic, social, cultural and economic factors at play in determining food choice [22]. Remote Australian communities are largely dependent on an imported food supply. There are approximately 175 stores, each servicing populations of over 100, operating in remote Indigenous communities in Australia [23]. The community store provides much or most of the local food supply [24,25]. The food distribution chain for many remote Indigenous communities extends over thousands of kilometres and is vulnerable to disruption by seasonal weather conditions and associated events such as extended road closures [26]. In some cases food delivery is only by fortnightly barge and/ or aircraft [23]. These are factors which drive the price of food up to almost 50% higher than that in metropolitan centres [27].

The history of food supply systems is likely to have a continuing influence on food supply and purchasing patterns. It was not until the late 1960s and early 1970s, after dismantling of a government ration system and introduction of a cash economy, that Aboriginal people in remote communities had the means to independently purchase their food. In the early days of the community “trade store”, the range of food was very limited with few perishables and fresh produce available, unless grown or hunted and collected locally. Access to a wider range of foods has increased with improved transport routes and infrastructure and increasing consumer demand. Living conditions for residents of these communities have had a similar slow development, with overcrowding common and many houses and household infrastructure important to food preparation sub-standard and in a state of disrepair [28]. The history of poor access and uptake of to formal education has resulted in low literacy and educational levels, which also impact on opportunities for exposure to nutrition education and promotion, and lack of employment opportunities contribute to maintaining community income at a generally low level [2].

Despite these challenges, remote communities have good potential to positively influence dietary patterns, due to their relative size, collective sense of community, strong historical traditional structures of authority, and store ownership arrangements [26]. Improvements in biochemical dietary markers and reduced risk in cardiovascular disease have been demonstrated in two remote Aboriginal Australian communities in association with community-led initiatives which included improving the availability of healthy food in the store and providing individual level feedback on health status and dietary messages [29,30]. Similar initiatives have occurred in the remote community setting to promote healthy eating, including store shelf-talkers, cooking demonstrations, and removal of top selling sugar sweetened beverages [31]. The effect of these however on purchasing is largely not documented.

Further to these community-level initiatives, the Australian government instigated a community store licensing regime in 2007 in the Northern Territory (NT), where stores must demonstrate compliance with a set of minimum standards which include stocking of a range of essential food items, a pricing policy, and retail practices that support healthy eating [32]. This has resulted in tighter government control over retail operating practices in largely non-competitive environments where historically greater opportunity for consumer exploitation existed. The two major not-for-profit NT store associations (the Arnhem Land Progress Aboriginal Corporation (ALPA) and Outback Stores (OBS)) servicing in total 38 of the around 90 remote stores in the NT have also instigated various food subsidy strategies to reduce the cost of healthy food [33,34]. Currently ALPA applies a no freight charge to fresh fruit and frozen, tinned and dried vegetables, fresh milk, yoghurt and cheese. Similarly, OBS does not apply a freight component to fresh fruit and vegetables and since 2010, has made bottled water available at a reduced price in all its stores. Similar initiatives have been instigated by some independent store operators and other store associations in remote Indigenous Australia.

Despite such nutrition-related initiatives occurring in remote Indigenous Australia, most are not evaluated or designed to provide robust evidence of effect. This has contributed to the paucity of evidence about the most effective means of supporting healthy eating behaviour. The inferior health of Indigenous Australians relative to other Australians, and the role of poor nutrition in the health disparity presents a compelling reason to identify cost-effective interventions for nutrition improvement. This study provides the opportunity to examine the effectiveness and cost-effectiveness of evidence-based strategies that have the potential to positively influence food and drink purchasing in the context of a socio-economically disadvantaged population.

### Study aim

**Figure 1:**
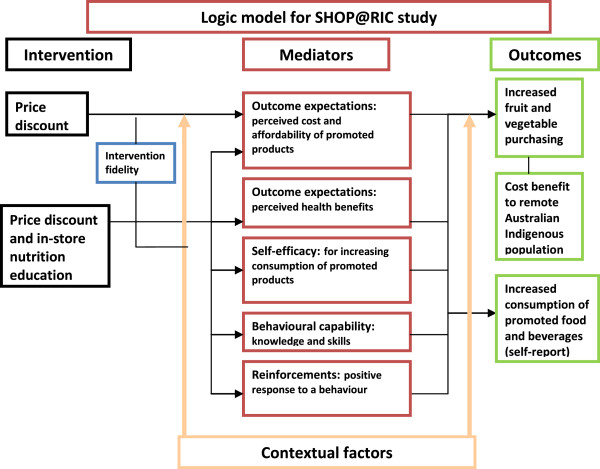
Study logic model.

The study tests the hypothesis that, over a six-month intervention and at six-months post-intervention, there will be a beneficial impact of (i) a 20% price discount and (ii) the addition of in-store nutrition education to the price discount intervention on the primary outcome measure of per capita daily weight (grams) of fruits and vegetables combined purchased through the community store, as well as on secondary outcome measures relating to the purchase of food and beverages and to the nutrient composition of purchased foods through the community store. The effect of the intervention will be measured via store point-of-sale data. In these remote communities where there is a single food store, point-of-sale data can provide a reliable and objective indicator of community level dietary intake [25,35]. A secondary aim of SHOP@RIC is to assess cost-effectiveness of the strategies to determine if the strategies represent good value-for-money in relation to disability adjusted life years (DALYs) saved over the lifetime of the community’s population. Overall food purchases and total diet/ energy intake will be assessed to document any unintended consequences of the strategies. An assessment of the influence of contextual factors, proposed mediators of behaviour change such as self-efficacy and perceived affordability of healthy food, and intervention fidelity will be conducted to aid interpretation of study findings and the understanding of mechanisms of behaviour change (Figure 1). This protocol relates to the primary aim of the study to assess effect of the price discount with or without the in-store nutrition education strategy on the primary and secondary outcome measures.

## Methods

### Setting

Aboriginal and Torres Strait Islander people represent 2.5% of the Australian population [36]. Twenty-four percent of Indigenous people live in remote/ very remote areas of Australia in small settlements commonly referred to as communities and/or homelands. These communities vary in size from an extended family group to over 2500 people, with most having less than 1000 people. Very remote areas are defined by geographic distance which imposes a severe restriction on accessibility to a range of goods and services [37]. These communities are also considered to be socio-economically disadvantaged according to the Socio-Economic Indexes for Areas (SEIFA index) based upon aggregated social and economic information collected through the national Australian population census [38]. Indigenous people represent 30% of the NT population and 72% live in remote or very remote areas.

The NT covers an area of 1,352,176 square km (which is equivalent to France, Italy and Spain combined). The majority of stores in the NT are owned and controlled by the Indigenous community as not-for-profit organisations and are governed by a board of directors, generally referred to as a store committee [26]. These committees comprise community leaders and/or community elders. Commonly, a non-Indigenous manager is appointed privately or by a consultancy firm contracted to manage the store [26].

The proposed study represents a collaboration with the two major store associations in the NT, ALPA and OBS and the respective store-owners. In the NT, ALPA manages 16 stores and OBS manages 22 stores.

### Design

The SHOP@RIC study is a multiple baseline (stepped wedge) randomised trial [39,40]. Twenty consenting communities have been randomly grouped into sets of 4 communities. Because of the logistical difficulty of commencing the price discount intervention and nutrition education strategies simultaneously in all communities, each set of four communities was randomly allocated to initiate the intervention at one of five possible time-points (the first set commenced in June 2013), spaced two-months apart. A 20% price discount on fresh and frozen fruit and vegetables, water and diet soft-drinks will be applied for a period of six months in each of the twenty communities and co-exist with other food-related promotions that may occur during this period. All community residents will have access to the intervention. Half the communities (i.e., ten stores; approximately one ALPA and one OBS store in each of the five sets) have been randomly allocated to receive the combined intervention – price discount and in-store nutrition education strategy. The study will apply a six-month intervention and pre-, post-, and six-month follow-up assessments of intervention effect.

#### Process evaluation - intervention confounders, mediators and intervention fidelity

Descriptive data on potential covariates (referred to in Figure 1 as contextual factors – these include factors such as frequency of food delivery, population movement, functioning of store equipment, retail practice) will be collected weekly for all communities through a structured survey administered over the course of the study. A before-and-after sub-study among a cohort of 150 community members recruited from five communities (comprising one community receiving the combined intervention in each of the five store sets), will examine hypothesised mediators of intervention effect on self-reported intake of fruit, vegetables, water, diet soft-drinks and sugar-sweetened soft-drinks. Qualitative and quantitative methods will be used to assess intervention fidelity, including an assessment of dose, reach and barriers and enablers to implementation.

### Theoretical framework

The strategy is informed by social ecological theory that proposes that behaviour is shaped by interaction between the individual and the environment [41] and that health promotion therefore requires both behavioural (e.g. nutrition education) and environmental strategies (e.g. price discount). It is also guided by Social Cognitive Theory (SCT) that asserts that individuals adopt new behaviours through social learning which involves outcome expectancies (i.e., consideration of the consequences of actions), observational learning, self-efficacy (i.e., confidence in performing a behavior), behavioural capabilities (i.e., skills and knowledge), and reinforcement [42]. These mediators may intervene in the process of learning and behaviour change. An understanding of these mediators is important in determining the most successful intervention elements and how they operate to change behavior. Figure 1 provides an overview of the program logic that underpins the relationship between the intervention, co-variates (contextual factors and the mediators of behaviour change), intervention fidelity, and outcomes (combined fruit and vegetable purchasing and intake).

Ethical approval has been granted by the combined NT Department of Health and Menzies School of Health Research Human Research Ethics Committee (ref: HREC-2012-1711), the Central Australian Human Research Ethics Committee (ref: HREC-12-13) and Deakin University Human Research Ethics Committee (ref: HREC-2012243).

### Eligibility and recruitment

#### Recruitment of stores

Twenty community stores (twelve OBS stores and eight ALPA stores) met the following criteria and were invited to participate in the study:

– Community store managed by either ALPA or OBS

– Community located within the NT

– Store located more than 20 kilometres from its nearest competitor

– Community population of 100 residents or greater

Initial contact was made with a representative of each of the community store boards and information provided on the study. This contact was facilitated by OBS and ALPA personnel and either an OBS/ ALPA representative alone or together with a member/s of the research team, visited each community. A study “story” was used to faciliate a discussion with store board representatives and to ensure comprehensive understanding of the study and study protocol. Through this process, research agreements, outlining the responsibilities of all parties (ALPA/OBS, store owners and Menzies School of Health Research), were signed. Store-owners were asked to assist with the recruitment of community-based research assistants. These personnel will be trained to assist with all community-level study activities, and invited to participate in a workshop at the end of the study to interpret study findings and plan for research dissemination.

#### Recruitment of participants to sub-study for assessment of mediator measures

Approximately 40 adults will be recruited to participate in each of the five sub-study subset communities. It is expected that an initial sample of 40 will result in a final sample of at least 30, allowing for attrition over the study period (minimum of 150 from across five communities). On a visit to each household randomly selected to participate, an adult meeting the eligibility criteria (community resident, plans to reside in the community for 12 months, over 18 years, purchases food from the community store, and is the primary shopper) will be invited to participate in a survey and requested to provide written consent following explanation of purpose of the sub-study and of the interview. On completion of the survey at each of the three time points, a AUD20 gift of fruit and water will be provided to each participant. Feedback of key messages from the sub-study will be provided to participants at the end of the study, based on aggregated data.

### Randomisation

We pre-determined that each of the store sets would consist of mostly two ALPA stores (of which one would be randomly allocated to receive the nutrition education strategy) and two OBS stores (of which one would be randomly allocated to receive the nutrition education strategy). We randomly selected a store from each of the ALPA group of stores (contained in an opaque envelope) and OBS group of stores (contained in an opaque envelope), and in turn continued to consecutively allocate stores to the fixed store set framework, starting with the first store slot in the first store set, until all stores had been allocated. It was decided prior to randomisation when the price discount would begin for each set of stores.

### Blinding

Study stores cannot be blinded to the timing of the intervention in their community and its duration. Research staff administering the intervention will also not be blinded, as they will be required to liaise/communicate with stores through the course of the intervention. This may impact on self-reported dietary intake data collected at the individual level as part of the mediation sub-study. The data management team will be blinded to intervention allocation. Data will be linked to a store identifier by an independent data administrator, immediately prior to sending to the statistician. The statistician will have no contact with communities or their information before the final data is ready for analysis, and will not know which ten stores received the education strategy and which did not when analyzing the data (as the meaning of the education strategy codes will be concealed until analysis is complete).

### Interventions

#### Price discount

Twenty percent is the same level of discount as that being trialled by the SHELf study [18] and higher than the 12.5% used for the NZ SHOP study [13]. The discount will be applied electronically at point-of-sale on all discounted products. A reduction of this size is considered unlikely to lead to the unintended consequence of increasing purchase of high energy nutrient poor foods that a higher price reduction might encourage [18], although such a response to a price discount has not yet been demonstrated [16]. The price discount will be promoted in-store through a poster at front-of-store, shelf-labelling and price ticketing on targeted products showing both the “usual” and discount price. The discount will be applied electronically at the point-of-sale. The purchase receipt will indicate where a price discount has been applied.

#### In-store nutrition education strategy

Development of the in-store nutrition education strategy has been informed by SCT [42] and focuses on the elements of SCT considered important in mediating behaviour change: influencing people’s perceptions of healthy food and food affordability (outcome expectancies), building people’s confidence and skills to make healthy food choices (self-efficacy and behavioural capabilities), modelling behaviour (observational learning) and reinforcing positive behaviour through providing incentives and rewards (reinforcement) [18]. The development of the strategy has also drawn on key aspects of the context of eating behaviour in remote Indigenous communities, including the importance of family and relationships, the autonomy granted to children, the role of elders in knowledge transfer, and the pivotal sense of freedom to choose. The nutrition education strategy was designed to be low cost, require few resources and to include a range of activities commonly implemented in remote community stores.

The reduced cost and benefits of consuming more of the healthy foods will be promoted through the nutrition education strategy, and suggestions and activities provided on how to increase fruit and vegetable intake and substitute water or diet soft-drinks for sugar-sweetened soft-drinks. Six themes have been developed with a supporting set of activities to be implemented over the six-month intervention period including posters, activity sheets, cooking demonstrations and taste-testing activities and competitions.

### Study outcomes

The primary outcome measure is the per capita daily weight (grams) of combined fruit and vegetables purchased through the community store. Secondary outcome measures are: 1) per capita daily weight (grams) of diet soft-drinks; water; fruit; and, vegetables; 2) percent total sugars (grams) to energy (kJ); 3) sodium (mg) per 1000 kJ of energy; 4) proportion of total sugar-sweetened soft-drinks (grams) to total combined fruit and vegetable (grams); and, 5) proportion of healthy foods to unhealthy foods (using pre-defined criteria), purchased through the community store (Table 1).

**Table 1:** Summary of study measures

**Measures**	**Baseline**	**Post intervention**	**Follow-up**
**Primary and secondary outcomes**			
***Store level***			
Combined fruit and vegetable purchasing (grams/person/day)	✓	✓	✓
Water, diet soft-drink, fruit and vegetable purchasing (grams/person/day)	✓	✓	✓
Percent total sugars (grams) to total energy (kJ) purchased	✓	✓	✓
Sodium (mg) per 1000 kJ of total energy purchased	✓	✓	✓
Proportion of total sugar-sweetened soft drinks purchased (grams) to total fruit and vegetables purchased	✓	✓	✓
Proportion of healthy food to unhealthy food purchased	✓	✓	✓
**Other outcomes to measure impact on total diet**			
Totals of each of dietary energy (kJ), sodium (mg) and sales ($) and sub-totals for each of 20+ food groupings	✓	✓	✓
Proportions per 1000 kJ of energy for each of fruit and vegetables (grams), sugar sweetened soft drinks (grams), healthy foods (grams) and unhealthy foods (grams)	✓	✓	✓
Proportion of healthy foods to total foods, unhealthy food to total foods and diet soft-drinks and water to total beverages, using a total energy (kJ), sales ($) and weight (grams)	✓	✓	✓
**Mediation sub-study outcomes**			
Fruit consumption	✓	✓	✓
Vegetable consumption	✓	✓	✓
Water consumption	✓	✓	✓
Diet and sugar sweetened soft-drink consumption	✓	✓	✓
**Process evaluation**			
***Contextual factors***			
Population movement	✓	✓	✓
Positioning of food and beverage products in-store	✓	✓	✓
Price and price ticketing practice	✓	✓	✓
Range of food and beverages available for purchase	✓	✓	✓
Provisioning of food and beverages to other services/organisations	✓	✓	✓
Community income	✓	✓	✓
Store policy	✓	✓	✓
Product promotion	✓	✓	✓
Store infrastructure	✓	✓	✓
Delivery of food and beverages	✓	✓	✓
Store management and staffing/ community social issues	✓	✓	✓
***Mediation sub-study***			
Outcome expectations of perceived affordability of fruit and vegetables	✓	✓	✓
Outcome expectations of perceived benefits of fruit and vegetables	✓	✓	✓
Self-efficacy for increasing fruit and vegetable consumption	✓	✓	✓
Self-efficacy for drinking water and reducing sugar sweetened soft drink consumption	✓	✓	✓
Food security	✓	✓	✓
Personal preferences	✓	✓	✓
Barriers to consuming more fruit and vegetables	✓	✓	✓
**Intervention fidelity**			
Intervention fidelity		✓	
Dose and reach of nutrition education strategy activities		✓	
Perceived barriers and enablers to intervention implementation (price discount and combined strategy)		✓	

Other dietary measures to be described and to assess impact of the strategies on the overall diet include a range of per capita per day measures: i) totals each of dietary energy (kJ), sodium (mg) and sales ($), and subtotals each of fruit, vegetables, diet soft-drinks, water and up to 20 other food groupings (derived from AUSNUT categorisation [43]); ii) proportions per 1000 kJ of energy for each of fruit and vegetables, sugar-sweetened soft-drinks, pre-defined healthy foods and unhealthy foods; and, iii) proportion of healthy foods to total foods, unhealthy foods to total foods and diet soft-drinks and water to total beverages using each of total energy (kJ), sales ($) and weight (grams).

### Data collection

#### Outcome measurements

Assessment of intervention effect on the primary and secondary outcomes will be made via store point-of-sale data collected over the course of the study. Point-of-sale reports by weekly (seven-day) periods will be generated for each store by ALPA and OBS and received electronically by the research team. These reports will be uploaded into a purpose-built Microsoft Access database [44] and linked with nutrient composition data [43,45]. Australian Bureau of Statistics data from the 2011 national census will be used for study population estimates [46].

#### Process evaluation

An electronic survey tool has been developed to collect data from store managers and community key informants on factors identified by experts to influence food and beverage purchases including (Table 1):

• Store retail practices

• Community events or activities

• Population movement

• National or global events/ activities

Data on these factors will be collected for each of the study communities throughout the course of the study commencing in May 2013. Information on these contextual factors will be used to identify potential analytical covariates and to aid interpretation of impact of the intervention to the outcome measures. To examine proposed mediators of behaviour change, data will be collected on mediator measures and self-reported consumption of fruit, vegetables, water, diet soft drinks and sugar-sweetened soft drinks. These data will be collected pre- and immediately prior to the end of the intervention and at six-months follow-up, using survey items adapted from previously published scales [18] after extensive pilot-testing to make them more appropriate to the cultural context of the study population. Additional data will be collected to assess intervention i) fidelity; ii) dose and reach; and, iii) perceived barriers and enablers to implementation. This will involve the collection of observational data including in-store spot checks; documentation of activities implemented; and interviews with key stakeholders (including the store manager, store staff and key community informants). A summary of all study measures is shown in Table 1.

### Analyses

#### Primary and secondary outcome measurements

Mixed models will be used to analyse the store point-of-sale data; random effects will include the store; fixed effects will include the step (of 8 weeks in length which corresponds to the “lag” in the roll-out of the intervention across sets of stores), the phase (baseline, intervention, post-intervention) and the in-store nutrition education strategy. Additional covariates (the contextual factors - such as distance to competing food outlet, community level income, population size) will be described and tested and may be introduced as additional fixed effects. It is expected that there will be some additional correlation within each store and a variance-covariance model (VCVM) for this will be identified prior to the formal testing of the fixed effects and other covariates. Mediation analyses will be undertaken using the MacKinnon method [47]. Descriptive statistics (for quantitative data), content and thematic analysis (for qualitative data) will be used as appropriate to analyse data on intervention fidelity. This information will be used to aid interpretation of results of the outcome measures.

#### Cost-effectiveness evaluation

The cost-effectiveness analysis will combine data on the effectiveness and cost of the intervention to determine whether it represents value-for-money. It will be measured against usual practice during the six-month period prior to intervention. Resource use and costs associated with the intervention will be prospectively measured. In addition to the ‘trial-based evaluation’ (costs and outcomes exactly as per the trial), a ‘modelled economic evaluation’ will be conducted which extends the target population (all remote indigenous communities), time horizon (rest of life of the cohort) and decision context. The incremental change in the purchasing of fruit and vegetables and of diet soft-drinks and water associated with the intervention will be converted to incremental cost-effectiveness ratios expressed as $ per Disability-Adjusted Life Years (DALYs) saved. Standard discounting will be applied to both costs and outcomes. Simulation-modelling using the @RISK software package will be employed to calculate 95% uncertainty intervals (median, 2.5 and 97.5 percentiles) around the epidemiological probabilities and cost and outcome estimates.

### Sample size

#### Primary outcome measurement

The power calculations were based on 1000 simulated datasets that each mimicked the design of the study. Data were simulated that resembled the first 37 weeks of baseline, combined-fruit-and-vegetable store point-of-sale data, and also assumed that the interventions would be effective. Specifically, log-normally distributed data were simulated assuming mean 8-weekly sales of fruit and vegetables (expressed as grams per day) per capita of 4.77 (on the log scale) and a latent between-store SD of 0.48. Other assumptions included were that residuals were independent and identically normally distributed with mean 0 and SD 0.16 (all on the log scale), and that (geometric) mean sales in a store (on the original scale, and in the absence of residual noise) will be 20% higher in a store when the price discount is on, and 20% higher again if a store receives the nutrition education.

Analysing these datasets by utilising data not beyond the end of the intervention period, log-transforming the data, and then using a mixed model [40] with random effects for store, and fixed effects for each 8-week time period, as well as for the two strategies, we estimate that analysis of combined fruit and vegetable point-of-sale data collected from a sample of 20 stores in our study has approximately 95% power to detect a common 20% change in (geometric) mean sales in the stores due to the price-discount intervention (at the 5% significance level), and approximately 97% (power to detect a 20% change due to the nutrition education strategy per se. A 20% increase in fruit and vegetable sales combined equates to an approximate increase of 30g of fruit and vegetables per person per day.

## Discussion

This study provides an opportunity to rigorously examine both the effect and cost-effectiveness of a price discount alone and in combination with nutrition education. It reflects an excellent collaborative effort between researchers and store-owners and major retailers in the remote Australian landscape to promote healthy eating and to provide empirical evidence to inform food pricing policy. Aboriginal community leaders have identified the high cost of food as a barrier to improving community level diet and addressing the health disparity between Indigenous and non-Indigenous Australians. Growing evidence indicates that the cost of food impacts on poor health outcomes especially for people with a limited income [48]. Evidence indicates from general population studies that price discounts tend to be effective in positively modifying food purchasing [13]. There is little such evidence relating specifically to socio-economically disadvantaged populations. Evidence on the role of nutrition education in modifying food purchases among socio-economically disadvantaged populations is also limited. The way in which remote community stores operate and the quality of food they provide are considered critical by community leaders and to the Australian Government’s effort to improve the health of Indigenous people living remotely. Approaches to increasing spending on fruit and vegetables and reducing spending on sugar-sweetened soft drink are currently being considered.

The proposed study will provide evidence critically needed to contribute to the cost-effective reversal of the epidemics of poor nutrition and associated adverse health outcomes in the remote Indigenous population. It will provide evidence on the role of price discount and in-store nutrition education interventions to improve diet and health outcomes for socially disadvantaged populations in general.

## Abbreviations

ALPA: Arnhem Land Progress Aboriginal Corporation; NT: Northern Territory; OBS: Outback Stores; SEIFA: Socioeconomic index for areas; SHOP@RIC: Stores healthy options project in remote Indigenous communities; SCT: Social cognitive theory.

## Competing interests

The authors declare that they have no competing interests.

## Authors’ contributions

JB conceived of and designed the study, developed the intervention and evaluation components, and drafted the manuscript. MF contributed to study conception, design, intervention development and development of survey questionnaire design. SL contributed to intervention design and development and evaluation components. KB contributed to study design and survey questionnaire design and development. MM and AM contributed to study design and designed the economic evaluation. EM contributed community engagement and remote community retail operations expertise and advised on study processes. MC contributed to study design and the analysis plan. CNM contributed to study design. AL, RB and KO contributed to study design and refinement of study methods. All authors contributed to manuscript preparation and read and approved the final manuscript.

## Pre-publication history

The pre-publication history for this paper can be accessed here:

http://www.biomedcentral.com/1471-2458/13/744/prepub

## References

[B1] Vos T Barker B Begg S Stanley L Lopez AD Burden of disease and injury in aboriginal and Torres Strait Islander Peoples: the Indigenous health gap Int J Epidemiol 2009 38 470 477 10.1093/ije/dyn240 19047078

[B2] Australian Institute of Health and Welfare The health and welfare of Australia's Aboriginal and Torres Strait Islander people, an overview 2011. Cat no: IHW 42 2011 Canberra: AIHW http://www.aihw.gov.au/WorkArea/DownloadAsset.aspx?id=10737418955&libID=10737418954

[B3] Brimblecombe JK Ferguson MM Liberato SC O'Dea K Characteristics of the community-level diet of aboriginal people in remote northern Australia Med J Aust 2013 198 380 384 10.5694/mja12.11407 23581959

[B4] Lock K Pomerleau J Causer L McKee M Ezzati M, Lopez A, Rodgers A, Murray C Low fruit and vegetable consumption Comparative quantification of health risks:global and regional burden of disease attributable to selected major risk factors 2004 Geneva: Wolrd Health Organisation 597

[B5] SIGNAL and the National Vegetables and Fruit Coalition Eat more vegetables and fruit: The case for a five-year campaign to increase vegetable and fruit consumption in Australia. Part 1: Business Case 2002 Canberra: SIGNAL

[B6] Schulze MB Manson JE Ludwig DS Colditz GA Stampfer MJ Willett WC Sugar-sweetened beverages, weight gain, and incidence of type 2 diabetes in young and middle-aged women JAMA 2004 292 927 934 10.1001/jama.292.8.927 15328324

[B7] Chen L Appel LJ Loria C Lin PH Champagne CM Elmer PJ Reduction in consumption of sugar-sweetened beverages is associated with weight loss: the PREMIER trial Am J Clin Nutr 2009 89 1299 1306 10.3945/ajcn.2008.27240 19339405 PMC2676995

[B8] Laville M Nazare JA Diabetes, insulin resistance and sugars Obes Rev 2009 10 Suppl 1 24 33 19207533 10.1111/j.1467-789X.2008.00562.x

[B9] Rowley KG Iser DM Best JD O'Dea K Leonard D McDermott R Albuminuria in Australian aboriginal people: prevalence and associations with components of the metabolic syndrome Diabetologia 2000 43 1397 1403 10.1007/s001250051545 11126409

[B10] Maple-Brown L Cunningham J Celermajer DS O'Dea K Increased carotid intima-media thickness in remote and urban indigenous Australians: impact of diabetes and components of the metabolic syndrome Clin Endocrinol (Oxf) 2007 66 419 425 10.1111/j.1365-2265.2007.02749.x 17302878

[B11] An R Patel D Segal D Sturm R Eating better for less: a national discount program for healthy food purchases in South Africa Am J Health Behav 2013 37 56 61 10.5993/AJHB.37.1.6 22943101 PMC3433851

[B12] Eyles H Ni Mhurchu C Nghiem N Blakely T Food pricing strategies, population diets, and non-communicable disease: a systematic review of simulation studies PLoS Med 2012 9 e1001353 10.1371/journal.pmed.1001353 23239943 PMC3519906

[B13] Ni Mhurchu C Blakely T Jiang Y Eyles HC Rodgers A Effects of price discounts and tailored nutrition education on supermarket purchases: a randomized controlled trial Am J Clin Nutr 2010 91 736 747 10.3945/ajcn.2009.28742 20042528

[B14] An R Effectiveness of subsidies in promoting healthy food purchases and consumption: a review of field experiments Public Health Nutr 2013 16 1215 1228 10.1017/S1368980012004715 23122423 PMC3898771

[B15] Black AP Brimblecombe J Eyles H Morris P Vally H Dea O Food subsidy programs and the health and nutritional status of disadvantaged families in high income countries: a systematic review BMC Publ Health 2012 12 1099 10.1186/1471-2458-12-1099 PMC3559269 23256601

[B16] Waterlander WE de Boer MR Schuit AJ Seidell JC Steenhuis IH Price discounts significantly enhance fruit and vegetable purchases when combined with nutrition education: a randomized controlled supermarket trial Am J Clin Nutr 2013 97 886 895 10.3945/ajcn.112.041632 23446898

[B17] Blakely T Ni Mhurchu C Jiang Y Matoe L Funaki-Tahifote M Eyles HC Do effects of price discounts and nutrition education on food purchases vary by ethnicity, income and education? Results from a randomised, controlled trial J Epidemiol Community Health 2011 65 10 902 908 http://jech.bmj.com/content/65/10/902.abstract. Epub 2011 Feb 4 10.1136/jech.2010.118588 21296903

[B18] Ball K McNaughton S Ni Mhurchu C Andrianopoulos A Inglis V McNeilly B Supermarket Healthy Eating for Life (SHELf): protocol of a randomised controlled trial promoting healthy food and beverage consumption through price reduction and skill-building strategies BMC Publ Health 2011 11 715 10.1186/1471-2458-11-715 PMC3186753 21936957

[B19] Jensen R Miller N Do consumer price subsidies really improve nutrition? 2010 Cambridge: National Bureau of Economic Research. NBER working paper series 1 43

[B20] Glanz K Eriksen MP Individual and community models for dietary behavior change J Nutr Educ 1993 25 80 10.1016/S0022-3182(12)80969-1

[B21] Glanz K Basil M Maibach E Goldberg J Snyder D Why Americans eat what they do: taste, nutrition, cost, convenience, and weight control concerns as influences on food consumption J Am Diet Assoc 1998 98 1118 1126 10.1016/S0002-8223(98)00260-0 9787717

[B22] National Health and Medical Research Council Nutrition in Aboriginal and Torres Strait Islander Peoples: An Information Paper 2000 Canberra: Commonwealth of Australia 1 267 http://www.nhmrc.gov.au/_files_nhmrc/publications/attachments/n26.pdf

[B23] Council of Australian Governments National strategy for food security in remote Indigenous communities 2009 Canberra: Council of Australian Governments [http://www.coag.gov.au/sites/default/files/nat_strat_food_security.pdf]

[B24] Lee AJ Smith A Bryce S O'Dea K Rutishauser IH Mathews JD Measuring dietary intake in remote Australian aboriginal communities Ecol Food Nutr 1995 34 19 31 10.1080/03670244.1995.9991444

[B25] Brimblecombe J Mackerras D Clifford P O'Dea K Does the store-turnover method still provide a useful guide to food intakes in aboriginal communities? Aust NZ J Public Health 2006 30 444 447 10.1111/j.1467-842X.2006.tb00461.x 17073226

[B26] The Parliament of the Commonwealth of Australia. House of Representatives: Aboriginal and Torres Strait Islander Affairs Committee Everybody's Business: remote aboriginal and Torres strait islander community stores 2009 Canberra: Commonwealth of Australia [http://www.aph.gov.au/Parliamentary_Business/Committees/House_of_Representatives_Committees?url=/atsia/communitystores/report.htm]

[B27] Northern Territory Government Northern territory market basket survey 2012 Darwin: Department of health, Northern Territory [http://digitallibrary.health.nt.gov.au/prodjspui/bitstream/10137/560/2/NT%20MBS%20Report.pdf]

[B28] Torzillo PJ Pholeros P Rainow S Barker G Sowerbutts T Short T The state of health hardware in aboriginal communities in rural and remote Australia Aust N Z J Public Health 2008 32 7 11 10.1111/j.1753-6405.2008.00158.x 18290906

[B29] Rowley KG Daniel M Skinner K Skinner M White GA O'Dea K Effectiveness of a community-directed 'healthy lifestyle' program in a remote Australian aboriginal community Aust NZ J Public Health 2000 24 136 144 10.1111/j.1467-842X.2000.tb00133.x 10790932

[B30] Lee AJ Bonson AP Yarmirr D O'Dea K Mathews JD Sustainability of a successful health and nutrition program in a remote aboriginal community Med J Aust 1995 162 632 635 7603373 10.5694/j.1326-5377.1995.tb126048.x

[B31] Butler R Tapsell L Lyons-Wall P Trends in purchasing patterns of sugar-sweetened water-based beverages in a remote aboriginal community store following the implementation of a community-developed store nutrition policy Nutr Diet 2011 68 115 119 10.1111/j.1747-0080.2011.01515.x

[B32] Australian Government Stronger futures in the northern territory policy statement november 2011 2011 Canberra: Australian government. Department of Families, Housing, Community Services and Indigenous Affairs 1 11 [http://www.fahcsia.gov.au/our-responsibilities/indigenous-australians/programs-services/stronger-futures-in-the-northern-territory-0]

[B33] ALPA - The Arnhem Land Progress Aboriginal Corporation 2013 ALPA [http://www.alpa.asn.au/]

[B34] OUTBACK Stores. OBS 2013 [http://www.outbackstores.com.au/]

[B35] Brimblecombe J Liddle R O'Dea K Use of point-of-sale data to assess food and nutrient quality in remote stores Public Health Nutr 2013 16 7 1159 1167 http://www.ncbi.nlm.nih.gov/pubmed/23009766, http://journals.cambridge.org/action/displayAbstract?fromPage=online&aid=8927689. Epub 2012 Sep 25 10.1017/S1368980012004284 23009766 PMC10271512

[B36] Australian Bureau of Statistics 2076.0 - Census of population and housing: characteristics of aboriginal and Torres strait islander Australians, 2011 2010 Canberra http://www.abs.gov.au/ausstats/abs@.nsf/Lookup/2076.0main+features1102011

[B37] Australian Bureau of Statistics Information paper: ABS views on remoteness. ABS Cat. No. 1244.0 2001 Canberra: Commonwealth of Australia [http://www.ausstats.abs.gov.au/ausstats/free.nsf/0/FCC8158C85424727CA256C0F00003575/$File/12440_2001.pdf]

[B38] Australian Bureau of Statistics 2033.0.55.001 - Census of Population and Housing: Socio-Economic Indexes for Areas (SEIFA) 2011 Australia: ABS 4-4-2013. 8-4-2013. [http://www.abs.gov.au/websitedbs/censushome.nsf/home/seifa?opendocument&navpos=260]

[B39] Brown CA Lilford RJ The stepped wedge trial design: a systematic review BMC Med Res Methodol 2006 6 54 10.1186/1471-2288-6-54 17092344 PMC1636652

[B40] Hussey MA Hughes JP Design and analysis of stepped wedge cluster randomized trials Contemp Clin Trials 2007 28 182 191 10.1016/j.cct.2006.05.007 16829207

[B41] Stokols D Translating social ecological theory into guidelines for community health promotion Am J Health Promot 1996 10 282 298 10.4278/0890-1171-10.4.282 10159709

[B42] Bandura A Social foundations of thought and action: a social cognitive theory 1985 Prentice-Hall, INC: Englewood Cliffs, NJ, US

[B43] Food Standards Australia New Zealand (FSANZ) AUSNUT 2007 Database Files FSANZ [http://www.foodstandards.gov.au/science/monitoringnutrients/ausnut/pages/ausnut2007databasefi4061.aspx]

[B44] Brimblecombe J Keeping track of healthy foods: towards improving the nutritional quality of foods sold in community stores in remote Australia 2008 Darwin, NT: Menzies School of Health Research http://www.menzies.edu.au/page/Research/Projects/Nutrition/Remote_Indigenous_Stores_and_Takeaways_RIST_Keeping_Track_of_Healthy_Foods_Tool_Development/

[B45] Food Standards Australia New Zealand (FSANZ) NUTTAB FSANZ [http://www.foodstandards.gov.au/science/monitoringnutrients/nutrientables/pages/default.aspx]

[B46] Australian Bureau of Statistics 2011 Census quick stats 2012 Canberra: Commonwealth of Australia [http://www.abs.gov.au/websitedbs/censushome.nsf/home/quickstats?opendocument&navpos]

[B47] Cerin E Mackinnon DP A commentary on current practice in mediating variable analyses in behavioural nutrition and physical activity Public Health Nutr 2009 12 1182 1188 10.1017/S1368980008003649 18778534 PMC4207270

[B48] Khaw KT Wareham N Bingham S Welch A Luben R Day N Combined impact of health behaviours and mortality in men and women: the EPIC-Norfolk prospective population study PLoS Med 2008 5 e12 10.1371/journal.pmed.0050012 18184033 PMC2174962

